# A rare combination of closed fracture of right talar body Sneppen 2 with associated medial malleolus: A case report

**DOI:** 10.1016/j.ijscr.2019.10.010

**Published:** 2019-10-16

**Authors:** Ihsan Oesman, Ahmad Nugroho

**Affiliations:** Department of Orthopaedic & Traumatology, Cipto Mangunkusumo National Central Hospital and Faculty of Medicine, Universitas Indonesia, Jalan Diponegoro No. 71, Jakarta Pusat, Jakarta 10430, Indonesia

**Keywords:** Fracture, Talar body, Sneppen 2, Medial malleolus, Internal fixation, Tension band wiring

## Abstract

**Introduction:**

Ankle fractures are common, with the incidence of up to 174 cases per 100,000 adults per year. The talus, the second largest tarsal bone, has distinctive imaging characteristics and injury patterns. The combination of talar body fracture in sagittal plane along with medial malleolus fracture is an unusual pattern of injury and rarely reported in the literature.

**Presentation of case:**

We presented a case of nineteen-year old male with pain on the right ankle as a result from motorcycle accident twelve hours before admitted to Cipto Mangunkusumo Hospital. After the patient underwent close reduction and immobilization using back slab, patient then underwent open reduction surgery of the right ankle then fixated using plate and screw combined by tension band wiring (TBW) at the right medial malleolus.

**Discussion:**

Talar and Medial malleolus fracture was initially evaluated by physical examination and radiographic imaging and classified by Sneppen classification. After the diagnosis was established the patient underwent ORIF plate, screw and tension band wiring. In post-operative x-ray examination, there was a significant improvement in talocrural angle and medial clear space but same result in tibiofibular overlap and tibiofibular.

**Conclusion:**

Open reduction and internal fixation (ORIF) plate and screw combined by tension band wiring (TBW) is the right surgical treatment for patient with closed fracture of right talar body Sneppen 2 with associated medial malleolus fracture. The outcome of the treatment is excellent because the patient presented was able to fully recover to normal activity.

## Highlights

• Talar fractures are uncommon.
• Anatomic and stable reduction of talar fractures is of paramount importance.
• ORIF plate screw and ORIF TBW can be used to treat talar fracture.
• Good prognosis can be achieved by ORIF plate screw and ORIF TBW.

## 1 Introduction

Talar fracture is a very rare case. It represents less than 1% of human bone fracture and between 3%–6% of fracture in the foot, with the incidence of 3.2 per 100,000 and predominantly male [1,2]. About 7%–38% of talar fracture occur in the body of talus. According to Sneppen [3], talar body fracture is classified to five groups: compression (talocrural joint), shearing (coronal or sagittal), posterior tubercle, lateral tubercle and crush fractures.

Being an uncommon case, the evidence available is still scarce, making the treatment of this fracture has a special challenge. Other challenges of this fracture’s management are avascular necrosis of the talus and the surgical approach as we know that talus is difficult to visualize because of the shape of the tibiotalar articulation and the overhang of the anterior and posterior tibial plafond. Furthermore, this fracture is usually followed by high energy trauma, making other severe injuries are commonly present [1,2,4,5]. This case report has been presented according to SCARE guideline [6].

## 2 Patient information

**Fig. 1:**
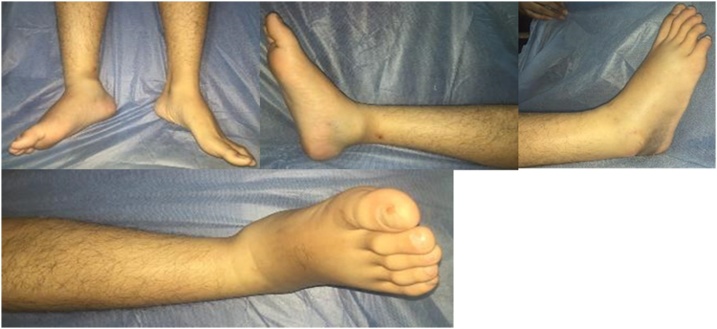
Clinical Appearance of the Patient’s Ankle.

**Fig. 2:**
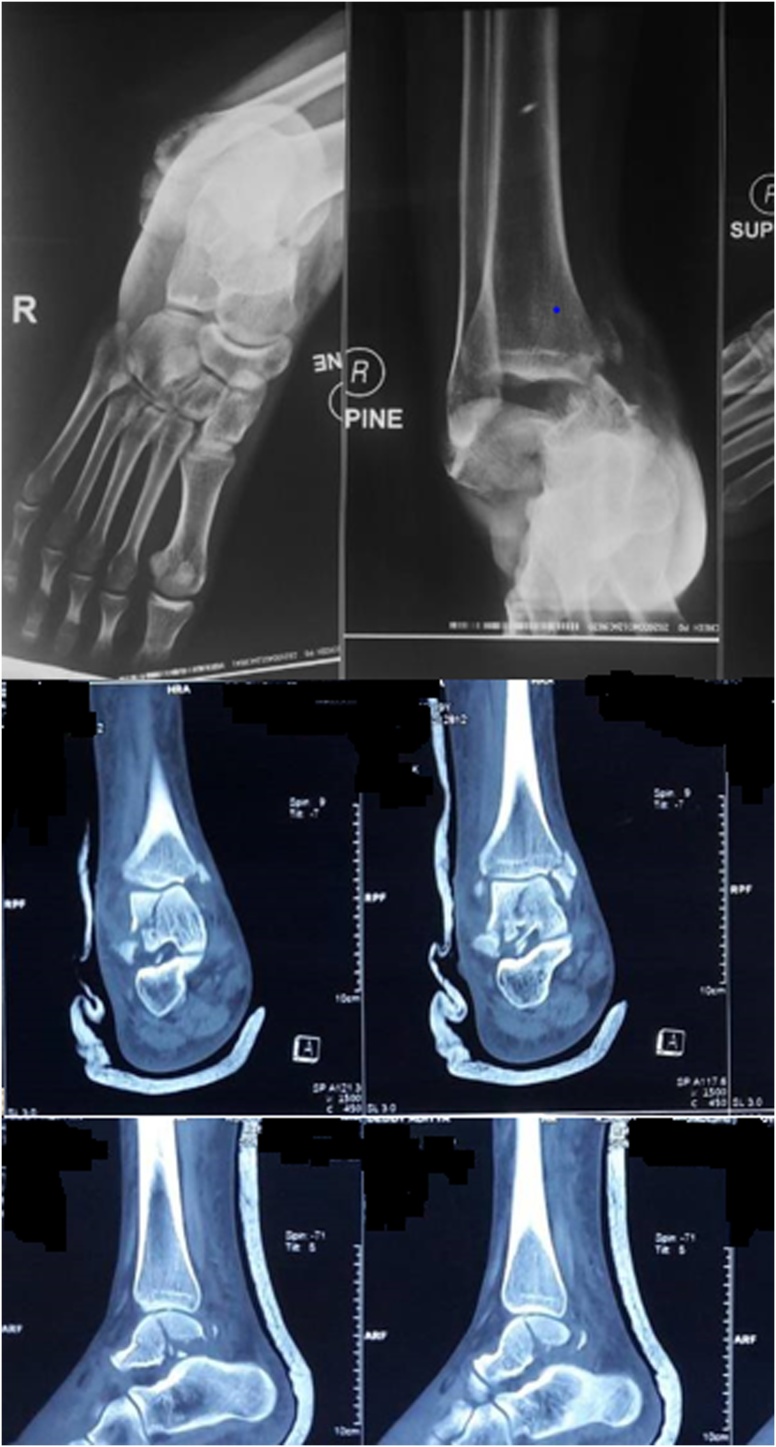
Right Ankle AP Lateral X-Rays and CT Scan showed fracture medial malleolus.

**Fig. 3:**
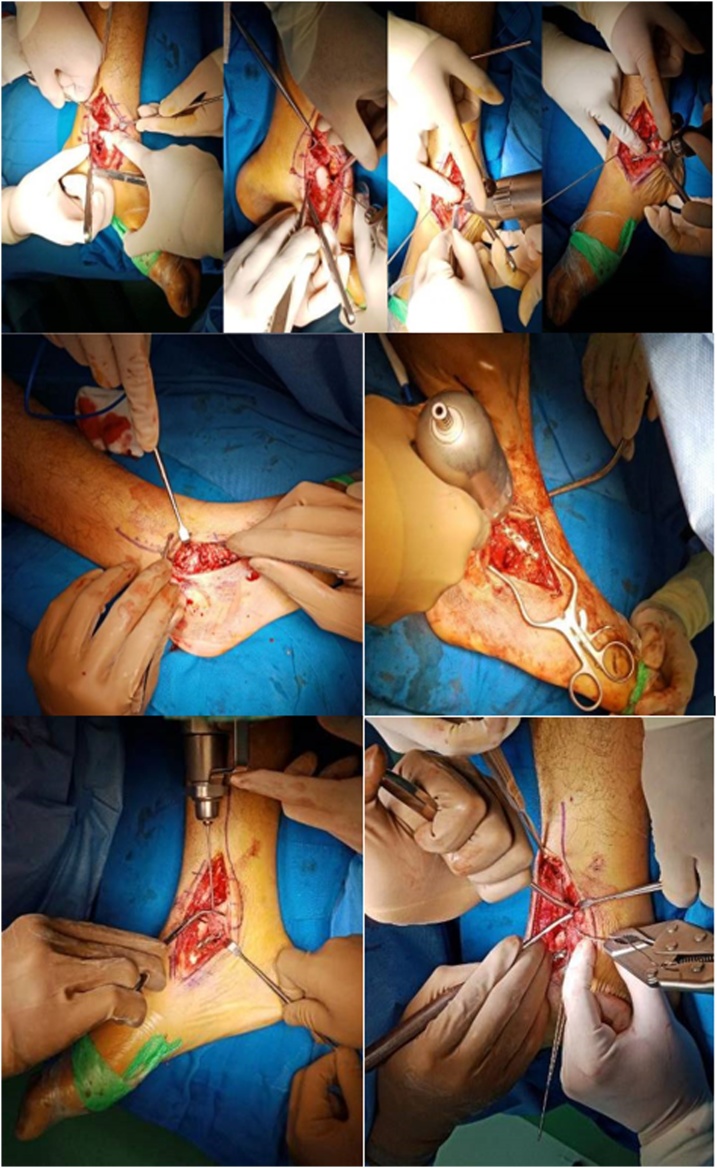
Intraoperative Procedure. Medial Expose, Wire Fixation, Plate Screw Insertion, Lateral Expose, Screw insertion, K Wire insertion, C wire Insertion.

**Fig. 4:**
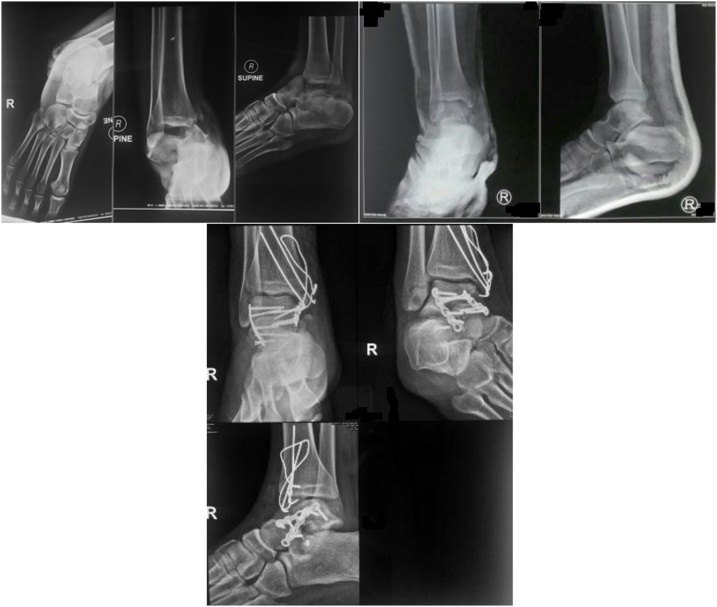
Ankle Imaging Initial, Post Closed Reduction, Post ORIF.

**Fig. 5:**
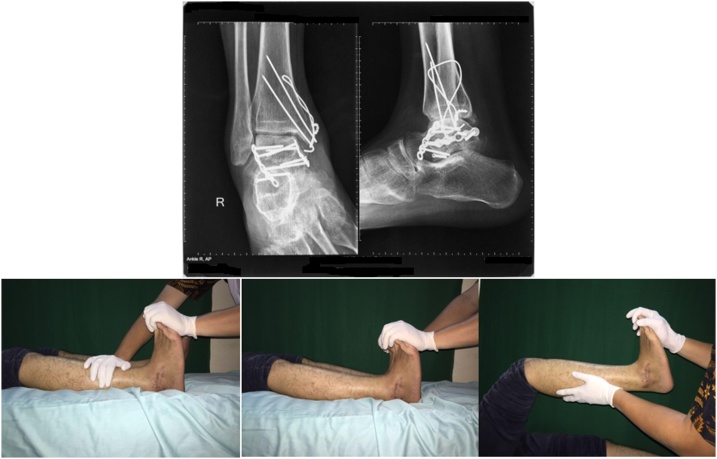
Radiological and Clinical Appearance 1 year postoperative.

We presented a 19 year old male that came to RSCM with chief complain of pain on the right ankle since 12 h prior to admission. This condition first appeared when he had a motorcycle accident. He hit the sidewalk while riding a motorcycle in high velocity. He was thrown away and landed with his right foot. The ankle was twisted to the lateral side. After the injury, patient felt pain on the right ankle (Fig. 1, Fig. 2, Fig. 3, Fig. 4, Fig. 5).

## 3 Clinical findings

The pain was exaggerated by movement. The ankle was swollen, but there was no open wound. Physical examination on right ankle revealed deformity, bruise, and hematoma with VAS 3. There was no wound or swollen, no abnormalities in neurovascular distal (NVD). Then we reduce the fracture in closed manner and immobilized the ankle by using a back slab in emergency room. The preoperative SF 36 and AOFAS score were 34 and 25, respectively.

## 4 Timeline

**Table:** 

Time	Symptom and Signs	Treatment
12 h prior to admission	Pain on right ankle. The ankle was swollen and deformed with bruise and hematoma. X-ray showed fracture on talar body and fracture on medial malleolus	Immobilization with back slab
12 h after admission	Pain on right ankle. Back slab was applied on lower extremity	ORIF plate screw and ORIF TBW

## 5 Diagnostic assessment

Normal blood test was found on the laboratory examination. On the radiology examination, AP Lateral projection of ankle x-ray, showed fracture on talar body and fracture on medial malleolus. The patient was diagnosed as close fracture of right talar body Sneppen 2 with associated medial malleolus fracture post Closed reduction and Immobilization using backslab (Table 1).

**Table 1:** Outcome of talar body fracture with specific treatment.

Ankle profile	Pre-operation	Post close reduction	Post ORIF	Normal Range	Note
Talocrural angle	58.05°	71.16°	70°	83 ± 4°	Better
Medial clear Space	7.7 mm	1.7 mm	3 mm	<4 mm	better
Tibiofibular overlap	13.5 mm	13.3 mm	13.3 mm	>10 mm	quite the same
Tibiofibular clear space	0 mm	0 mm	0 mm	<5 mm	same

## 6 Therapeutic intervention

He was planned to have open reduction and internal fixation (ORIF) with plateand screw and ORIF with tension band wiring (TBW). The approach used during surgery was anteromedial approach.

## 7 Follow up and outcomes

On 3rd day postoperative follow-up, we obtained good surgical wound, with no pus and no dehiscence. Post Op imaging revealed that there was an increase in the talocrural angle and medial clear space was in the normal range. The SF 36 and AOFAS scores were 40 and 28, respectively. Patient was rehabilitated according to the protocol of three phases of rehabilitation, in which maintaining range of movement in both ankle was performed in the first 6 weeks after surgery, bear partial weight in 6–8 weeks after surgery, and bear full weight after 12 weeks post-surgery. On 1 year postoperative follow up, we were obtained good outcome. Patient was able to fully recover to normal activity. The SF 36 and AOFAS scores at final follow up were 100 for both scores.

## 8 Discussion

Talar fractures are uncommon, only 0.1–0.85% of all fractures. Most of them are high energy injuries. More than 50% of all fractures involve talar neck, and up to 25% fractures involve body of talus [7]. The combination of talar body fracture in sagittal plane along with medial malleolus fracture is an unusual pattern of injury and rarely reported in the literature [8].

Talar body fractures are rare thus there is little information available to guide the management of these fractures. However, the outcome of talar body fractures is worse than neck fractures, because of the fact that neck fractures are considered extra-articular (involve only middle facet of subtalar joint) while body fractures are intra-articular (involve both tibiotalar and subtalar joints) [9]. There is also higher risk of avascular necrosis.

Sneppen et al. classified talar body fractures based on anatomic location into following types: Type A compression fracture, Type B coronal shearing fracture, Type C sagittal shearing fracture, Type D fracture in the posterior tubercle, Type E fracture in the lateral tubercle, Type F crush fracture. Later on, type B and type C were grouped into Sneppen Group 2 of talar body fracture [3]. Fracture of the talar body is caused by a force upon the talus that also causes fracture in ankle joint. Fractures of the talar body can be classified as compression injuries, shear fractures, fracture of the posterior process, fracture of the lateral process and crush fractures. They usually involve the articular surfaces, primarily the ankle joint and occasionally the subtalar joint [10]. Talar body Sneppen 2 or type B fracture or coronal shear fracture results from an axial load on a dorsiflexed foot in the setting of a high-level fall or motor vehicle accident [11,12]. This in accordance with the finding in our patient that he acquired the injury after suffered from accident. The most probable cause of mechanism of injury is dorsiflexion when he landed on the ground resulting in the shear fracture of the talar body. There are several approaches for surgical treatment of talar fracture, namely anteromedial, posteromedial, anterolateral, and posterolateral approaches. The most commonly used approach is the anteromedial approach. It was performed by making an incison medial to the tibialis anterior tendon. The fracture pattern and location will determine the choice of surgical approach. The main concern during surgical treatment is preservation of the blood supply [14]. As seen in study by Heather et al. [9], anteromedial approach was performed and showed satisfactory result. We also performed the surgery using anteromedial approach in this patient in order to preserve the blood supply for the foot and ankle.

Rehabilitation for patient with talar fracture consists of pre-and post-surgery rehabilitation. Pre-surgery rehabilitation is performed using temporary splint or cast in emergency room. This was in accordance with what we performed in emergency room after examination of the patient, where ankle was immobilized using back slab. Post-operative rehabilitation consists of three phases, namely phase I during first 1–6 weeks, phase II in 6–8 weeks, and phase III in 12–24 weeks. In phase I, patient is allowed for maintain motion in affected and unaffected joints to initiate early joint motion and control edema and pain. Patient was allowed to bear partial weight and increase the range of movement. In phase III, patient was allowed to bear the full weight [13]. The real shape of the injury in our case is a sagittal shear splitting talar body and medial malleolus in oblique direction from posteromedial to anterolateral. The mechanism of injuries is not clear but is expected to be a combination of dorsiflexion with axial compression, along with a supination element which is the cause of the medial malleolus fracture.

CT scan should be done to recognize the fracture pattern and amount of displacement and comminution. Open reduction and internal fixation should correct displaced fractures. Taking care to preserve the remaining precarious blood supply and aiming for anatomic congruous reduction of articular surfaces to avoid complications.

Both medial and lateral approaches in combination allow complete view of the talar dome and for the reduction to be visualized from either side, to avoid any malreduction. Medial malleolus fracture along with talar body fracture in sagittal plane is a blessing in disguise as it may preserve some blood supply through its intact deltoid ligament branches of posterior tibial artery to talar body. Hence the risk of avascular necrosis of talus is less in associated medial malleolar fractures.

This is due to preservation of the deltoid ligament and the associated deltoid branch of the posterior tibial artery supplying the talar body [15]. Union of the fracture in such case is extremely slow as it depends on a new blood supply growing into the avascular bone [16], therefore the fracture needs protection for a long time and non-weight bearing is recommended for 3 months or until union has occurred. Post-traumatic arthrosis varies from 16 to 100% after talar body fractures [17]. Malunion can produce significant alteration in load across the ankle and subtalar joints and result in arthrosis.

Anatomic and stable reduction of talar body fractures is of paramount importance for obtaining a reasonable functional outcome [18]. The reported case should have a good prognosis as it was closed and underwent immediate operative reduction with early signs of revascularisation [19].

Our patient has good post-operative outcomes in term of ankle profile. Thus, we assume the aim of the surgical treatment is achieved. Always keep in mind that these fractures are slow to unite, hence to be kept in below knee cast for longer time and non-weight bearing to be followed till fracture unites with monitoring at regular intervals. Most of the previous studies have advised non-weight bearing for a minimum of 6–8 weeks and have reported good clinical outcome with mild symptoms on follow up [8].

There are some case reports with similar fractures that describe their treatment and outcomes. A.D. Mendonca et al. wrote about talar body fracture in both sagittal and coronal planes with intact neck, with medial malleolar fracture using anteromedial approach. The patient get to full recovery with no evidence of AVN at 6 months follow up after doing Non-weight bearing for 8 weeks [20].

K. Shah et al. reported a sagittal fracture of talar body with medial malleolar fracture. Talus fracture was undisplaced and discovered intra-operatively. Medial malleolus fixed from medial side. Talus fixed from lateral side, open or percutaneous not mentioned. Unfortunately, the outcome wasn’t mentioned [21].

Kailash Laxman Devalia et al. had a sagittal fracture of talar body with medial malleolar fracture. Underwent an anteromedial approach then non-weight bearing for 3 months, patient recover full ROM, with sclerosis of lateral fragment and maintained joint space at 14-year follow-up [16]. H. Saidi et al. presented a sagittal fracture of talar body with medial malleolar fracture. Using anteromedial approach followed by non-weight bearing for 3 months resulted in good outcome, painless ankle at 6 months follow up [22].

J. Isaacs et al. mentioned a talar body sagittal fracture and comminuted talar neck fracture, with medial malleolar fracture treated with dual medial and lateral approach. Followed by non-weight bearing for 7 weeks. The outcome was mild pain at 12 months; no AVN on radiographs, but mild secondary osteoarthritic changes in subtalar joint [23].

Aditya Mootha et al. got a sagittal fracture of talar body with medial malleolar fracture patient. Using posteromedial approach followed by non-weight bearing for 6 weeks gave a good outcome at 3 months with no radiological signs of AVN [24]. Atif Mechchat et al. also reported a sagittal fracture of talar body with medial malleolar fracture case. Treated using anteromedial approach followed non-weight bearing for 3 months. The outcomes was little pain, mild secondary arthritis at ankle, and good ROM at 14 months follow-up [19]. Comparing with all others report, our case has relatively better outcomes. The patient is able to do normal activity and the ankle function is fully recovered without any pain or sign of osteoarthritis after 6 months.

## 9 Conclusion

Open reduction and internal fixation (ORIF) plate and screw combined by tension band wiring (TBW) is the right surgical treatment for patient with closed fracture of right talar body Sneppen 2 with associated medial malleolus fracture. The outcome of the treatment was excellent because the patient was able to fully recover to normal activity.

## Funding

The authors received no financial support for the research, authorship, and/or publication of this article.

## Ethical approval

The ethical approval was not required for this case report.

## Consent

Informed consent had been obtained from the patient before the manuscript was written.

## Author’s contribution

Ihsan Oesman: study concept, data collection, data interpretation, and writing the paper.

Ahmad Nugroho: data collection, data interpretation and writing the paper.

## Registration of research studies

N/A.

## Guarantor

Ihsan Oesman.

## Provenance and peer review

Not commissioned, externally peer-reviewed.

## Declaration of Competing Interest

The authors certify that They have NO affiliations with or involvement in any organization or entity with any financial interest or non-financial interest in the subject matter or materials discussed in this manuscript.
